# Canine vector-borne diseases in India: a review of the literature and identification of existing knowledge gaps

**DOI:** 10.1186/1756-3305-3-28

**Published:** 2010-04-08

**Authors:** Puteri Azaziah Megat Abd Rani, Peter J Irwin, Mukulesh Gatne, Glen T Coleman, Rebecca J Traub

**Affiliations:** 1 School of Veterinary Science, The University of Queensland, Queensland 4072, Australia; 2 School of Veterinary and Biomedical Science, Murdoch University, Western Australia 6150, Australia; 3 Bombay Veterinary College, Maharastra Animal and Fisheries Sciences University, Parel, Mumbai 400012, India

## Abstract

Despite the combination of favourable climate for parasites and vectors, and large populations of stray dogs, information concerning the epidemiology, diagnosis and management of canine vector-borne diseases in India is limited. However, with the country's expanding economy and adaptation to western culture, higher expectations and demands are being placed on veterinary surgeons for improved knowledge of diseases and control. This review aims to provide an overview of the current state of knowledge of these diseases in India and identify existing knowledge gaps in the literature which need to be addressed. The available literature on this subject, although limited, suggests that a number of canine vector-borne diseases such as filariasis, babesiosis and ehrlichiosis are endemic throughout India, as diagnosed mostly by morphological methods. Detailed investigations of the epidemiology and zoonotic potential of these pathogens has been neglected. Further study is essential to develop a better understanding of the diversity of canine vector-borne diseases in India, and their significance for veterinary and public health.

## Review

India has a wide range of climatic zones, from montane (cold, wet alpine) and semi-arid regions to the wet tropics, which make it suitable for a diverse range of vectors and pathogens of medical and veterinary importance, whose transmission and geographical distribution are closely linked to regional temperature, rainfall and humidity [1]. Knowledge of parasitic diseases of companion animals in India remains incomplete, particularly outside the subcontinent, despite climatic conditions that are often conducive for the transmission of enteric and vector-borne parasitic infections.

India's dog population is estimated at 25 million and can be divided into four categories which can be defined as follows: pets (restricted and supervised); family dogs (partially restricted, wholly dependent); community dogs (unrestricted, partially dependent); and feral dogs (unrestricted, independent) [2]. Approximately 80% of the population fall into the latter three categories leaving over 5 million dogs within the 'pet' category. In a recent survey, 17% of households in India were reported to own a pet/domesticated dog [3]. Despite this, information available to veterinarians concerning the prevalence, epidemiology, diagnosis and management of canine vector-borne diseases (CVBD) and those of zoonotic concern is scarce [4]. This is not entirely unexpected in a country such as India where agriculture is the means of livelihood for about two-thirds of the work force and competency in animal husbandry/production animal medicine are rightfully emphasised in university veterinary curricula to meet the demands of India's agricultural/rural community. However, with one of the fastest growing economies in the world, India's increasingly affluent middle class is becoming increasingly accustomed to Western culture. This has resulted in changing attitudes towards companion animal ownership, with higher expectations and demands being placed on veterinary surgeons and the companion animal industry for improved knowledge of veterinary diseases and products for treatment and control [4,5].

The purpose of this paper is to present a summary of the extant literature pertaining to CVBD in India and to provide an overview of the current state of knowledge of these diseases. In order to achieve this, a detailed review was undertaken of the available literature within Indian veterinary and animal science journals, many of which are not indexed in the internationally-accessible scientific search engines.

### Filarial nematodes of dogs

The filarial nematodes are characterized by their tissue tropism and their dependence upon blood-feeding arthropod vectors for transmission [6]. The most commonly reported species in dogs are; *Dirofilaria immitis*, *Dirofilaria repens*, *Acanthocheilonema reconditum*, *Acanthocheilonema dracunculoides*, *Brugia malayi*, *Brugia ceylonesis *and *Brugia pahangi *[7-9]. *Dirofilaria immitis *is responsible for heartworm disease in dogs, yet microfilaraemia associated with other filarial infections is commonly detected in blood films of dogs in tropical countries, which theoretically necessitates specific identification of the filarial parasite in order to exclude the non-pathogenic species. This requires experienced personnel and it may be difficult to detect multiple infections with more than one species of filarial worm [10]. Despite the availability of published measurements of various microfilariae, the inaccuracy of morphological diagnosis was demonstrated by Rishniw and colleagues (2006) [8] when microfilariae initially identified as *A. reconditum *were later characterised as *D. immitis *by molecular methods. Both *D. repens *and *Acanthocheilonema *spp. develop into adult worms in the subcutaneous tissue resulting in skin nodules. Adults of *Brugia *spp. are usually recovered from the mandibular, retropharyngeal or axillary lymphatics. Most infections with *D. repens*, *Acanthocheilonema *spp. and *Brugia *spp. are of minimal veterinary clinical significance, however all canine filariae have the potential to infect humans and remain significant from a public health perspective.

*Dirofilaria immitis *infection in humans is very rare and usually associated with pulmonary lesions or radiological coin lesions of the lung. The significance of *D. immitis *infection is the potential for a radiological misdiagnosis of primary or metastatic lung tumour, leading to thoracotomy for open lung biopsy or wedge resection of the lung to obtain the correct diagnosis [11,12]. Sporadic cases of immature heartworms in unusual locations in the human body such as the eye [13], mesentery [14], cerebral artery [15], spermatic cord [16] and liver [17] have also been reported. *Dirofilaria repens *is a parasite of the subcutaneous tissue in dogs that can also accidentally infect humans, causing a condition referred to as subcutaneous dirofilariasis. It is considered to be a re-emerging zoonosis, transmitted by mosquitoes, endemic to Southern and Eastern Europe and Asia, particularly Sri Lanka [18], Malaysia [19] and India [20]. The distribution of human cases of subcutaneous dirofilariasis appears to mirror the distribution of canine cases [18,20]. Several genera of mosquitoes are competent vectors for *D. immitis *and *D. repens*, including *Culex*, *Aedes *and *Anopheles *[9,21]. *Acanthocheilonema reconditum *and *A. dracunculoides *rarely cause significant illness in dogs. Their importance lies in the fact that their microfilariae can be easily confused with those of *D. immitis *and *D. repens*. The adults from these species can be found in the body cavity and subcutaneous tissues of dogs. They are prevalent in the United States, Italy [22], Egypt [23] and Africa [24]. The intermediate host for *A. reconditum *are *Ctenocephalides *(flea) and *Heterodoxus *(lice) [25], and a biting fly, *Hippobosca longipennis *acts as intermediate host for *A. dracunculoides *[26].

*Dirofilaria *spp., *Acanthocheilonema *spp. and *Brugia *spp., have all been reported in India [27-29]. During one recent survey, post-mortem examination of 240 indigenous dogs at a local slaughterhouse (for dogs) in northeast India revealed 34% of dogs harboured heartworm infection [30]. The authors noted that among the heartworm-positive dogs, 35% had non-patent infections and none of the animals demonstrated overt clinical signs of disease on brief ante-mortem inspection. Both *D. immitis *and *D. repens *were isolated at post-mortem examination from 57% (4/7) and 14% (1/7) of dogs respectively in the central Indian state of Orissa [31]. Two recent surveys of microfilaraemic dogs in Kerala [20] and Karnataka States [28] in southern India, found only *D. repens *at a prevalence of 7% (n = 160) and 21% (n = 400) respectively. It is important to note however that these latter studies on *Dirofilaria *utilized morphological methods for diagnosis, which can be potentially misleading as microfilarial dimensions of both species of *Dirofilaria *often overlap. Moreover, it may be difficult to detect multiple infections with more than one species of filarial worm. Although minimally pathogenic in dogs, *D. repens *is zoonotic and a number of human cases of subcutaneous dirofilariasis in the medical literature of India have been reported in the same region [20]. Despite the limited number of surveys performed, veterinarians in India strongly believe that heartworm is confined to the northeast and *D. repens *to southern India. This assumption is debatable since competent mosquito vectors for *D. immitis *are present throughout central and southern India. For example, *Aedes albopictus *[32-34], a competent vector for *D. immitis *is present in Maharastra, Karnataka and Pondicherry [35], and heartworm is yet to be reported in dogs from these areas. Moreover, the tropical climate of these regions have average temperature ranges from 20°C in winter to 37°C in summer [36] and this provides a suitable environment for *D. immitis *development within the vector [37-39]. It is known that the development of *D. immitis *can also vary within mosquito species [40,41] and the genetic diversity of different strains of the same species could therefore be accountable for the variation observed. A case of human pulmonary dirofilariasis due to *D. immitis *however, was reported in Mumbai in 1989, adding further doubt to its currently accepted geographical distribution [42]. Studies into the effect of temperature on larval development of Dirofilaria have largely focused on D. immitis with less information available on D. repens [43].

At present, 1.3 billion people worldwide are at risk of lymphatic filariasis and about 120 million people in 83 countries are affected. Amongst them, 45.5 million live on the Indian subcontinent [44]. *Brugia malayi *is responsible for 10% of cases of zoonotic lymphatic filariasis in humans and is restricted to the tropics [45]. Although the main reservoirs are populations of leaf-eating monkeys (*Presbytis *spp.) [46], this filarial nematode has also been found to infect cats in Malaysia [47] and Thailand [48]. *Mansonia*, *Aedes*, *Culex *and *Armigeres *are four genera of mosquitoes that are able to transmit brugian filariosis [49]. In people, the disease may range from causing few clinical symptoms, or sufferers may experience acute manifestations such as fever, rashes, orchitis, lymphadenitis and lymphagitis that, if progressing to chronic infection, will lead to lymphoedema or 'elephantiasis' [6]. Another species, *Brugia pahangi*, has not been recognized in natural infections in humans but is able to infect humans experimentally [50]. *Brugia pahangi *was found mixed with other filariid species in 54.7% of dogs (n = 68) in Malaysia [51] and was isolated from 7.6% (n = 52) of cats in Thailand [52].

*Brugia ceylonensis *was first described from the lymphatics of dogs in Sri Lanka in 1962 [53]. In a Sri Lankan survey of 65 dogs, 44.6% were positive for microfilaria; of these, 62% and 7% had single infections with *D. repens *and *B. ceylonensis *respectively, while 31% had mixed infections with both species [54]. An adult *B. ceylonensis *was recently isolated from the conjunctiva of a person in Sri Lanka [55] raising public health concerns about the zoonotic potential of this canine filaria.

*Brugian *filariasis accounts for approximately 5% of lymphatic filariasis cases in India where over 40 million people are estimated to be infected [56]. Recently, based on immunodiagnostic testing, 16/75 (21.3%) microfilaraemic dogs were shown to harbour *B. malayi *[57]. The role of dogs (and cats) as reservoirs of brugian filariasis has important implications for parasite control strategies. If canine and feline reservoir hosts exist in these areas, a more inter-sectorial approach to control may be required in addition to the traditional use of mass drug administration programs advocated by the World Health Organization. The species of *Brugia *recovered from dogs in Kerala therefore requires confirmation using molecular diagnostic tools, as it is possible that the immunodiagnostic tests utilized to diagnose infection in dogs cross-react with other *Brugia *spp. [58].

### Canine tick-borne diseases

Babesiosis is an important disease of domestic and wild Canidae, caused by intraerythrocytic piroplasms of the genus *Babesia *and, potentially, *Theileria*. Historically, canine babesiosis has been attributed to infection with either *Babesia canis *or *Babesia gibsoni*. A wide range of clinical signs is reported for babesiosis, with the greatest severity in younger dogs, which may present to the veterinarian in a state of shock [59]. Lethargy is the most common symptom, followed by anorexia, pale mucous membranes, vomiting, amber to brown urine, splenomegaly, jaundice, weight loss, tachycardia and tachypnea.

Based on genetic data, vector specificity and variation of pathogenicity, three species of large *Babesia *have been identified. It is diagnostically important to differentiate between *B. canis*, *B. vogeli *and *B. rossi *[60,61] since the virulence, prognosis and response to treatment differ for each organism. *Babesia vogeli *is found worldwide and considered to be mildly virulent [62]. It usually results in transient haemolytic anaemia with a regenerative response (reticulocytosis) or a subclinical infection. *Babesia canis*, found in Europe, is considered to be virulent in dogs while *B. rossi*, reported only in Africa, is highly virulent, often causing peracute disease, resulting in hypoxia, hypotensive shock and disseminated intravascular coagulation and death before anaemia can develop [63]. *Babesia gibsoni *(Asian genotype), *B. conradae *(previously Californian genotype) and the *B. microti*-like species (also referred to as the Spanish isolate or *Theileria annae*) are each smaller piroplasm species that cause progressive haemolytic anaemia; *B. conradae *may produce the highest level of parasitaemia with more pronounced anaemia, higher fatality rates and be more likely to become recrudescent following treatment than *B. gibsoni *[64]. *Babesia *spp. are transmitted to dogs by a wide variety of ixodid ticks including *Haemaphysalis longicornis, H. leachi, Rhipicephalus sanguineus *and *Dermacentor marginatus *[65], although infection from blood transfusions [66], transplacental transmission [67], and direct transmissions through bite wounds [68] have been reported. Furthermore, subclinical infections by milder strains may simply remain dormant in a state of premunity, until such time as the animal is immunocompromised by unrelated disease or by iatrogenic drug administration.

The situation regarding canine babesiosis in India is far from clear. Only 0.1% of dogs (n = 5,832) in Chennai were found positive for *Babesia gibsoni *[69] microscopically. Other studies found 9% and 22% of dogs in Uttar Pradesh [70] and Assam [71] to be infected with *Babesia *spp., respectively, however it is unclear which genotypes of *Babesia *were harboured by these dogs. The pathogenicity of *Babesia *is known to vary in different regions of India and this may be due to variations in the species and strains present. It is likely that both *B. vogeli *and *B. gibsoni *are co-endemic in India and the ticks *Rhipicephalus sanguineus *and *Haemaphysalis longicornis *are the putative vectors, respectively.

*Ehrlichia *is an alpha-proteobacteria belonging to the family Ehrlichiaceae. In dogs, species that are able to produce infection are *E. canis *(tropical canine pancytopaenia), *E. ewingii *(canine granulocytic ehrlichiosis), and *E. chaffeensis *(Human monocytic ehrlichiosis) [10,72]. The one that most commonly affects dogs and causes the most severe clinical signs is *Ehrlichia canis*. The prevalence of E. canis is dependent on the distribution of the vector, Rhipicephalus sanguineus tick, which is widespread across tropical and subtropical regions [65].

A handful of studies investigating the prevalence of canine ehrlichiosis in India using conventional examination of stained blood smears have reported prevalences between 0.35% in Punjab [73] and 18.9% in Nagpur [74]. To date, only one study reported using a species-specific nested PCR and found 46/98 (50%) privately owned dogs in Chennai positive for *E. canis *compared to 19% by microscopy [75]. However, amplicons were not subjected to DNA sequencing to confirm results and information with regards to the clinical status of these dogs was not reported. It is unknown if other species of *Ehrlichia *or *Anaplasma *of veterinary or public health significance are present.

Canine hepatozoonosis is a systemic infection caused by the protozoan *Hepatozoon canis*. It is transmitted by ingestion of an infected dog tick, *R. sanguineus*, rather than tick bites [76]. The distribution of *H. canis *reflects the geographical distribution of its vector, which is present in Africa, southern Europe, Asia, Australia and the Americas [65]. The clinical spectrum of *H. canis *infection ranges from subclinical to severe, life-threatening disease [77]. *Hepatozoon canis *mainly infects the haemolymphatic tissue and blood cell-forming organs including the bone marrow, lymph nodes and spleen. Dogs with severe clinical disease show signs such as fever, inappetence, weight loss, anaemia, hyperglobulinaemia often resulting in hepatitis, pneumonia and glomerulonephritis associated with *H. canis *meronts. Co-infection of *H. canis *with other infectious agents such as *Ehrlichia, Leishmania *and parvovirus is common [78-80]. Immune suppression induced by an infectious agent or chemotherapy may reactivate pre-existing infections [79].

Canine hepatozoonosis has been reported in dogs in Mumbai associated with clinical signs of anaemia, thrombocytopenia, hepatitis, hyperglobulinaemia and elevate blood urea and nitrogen [81]. Information with regard to the immune status of these dogs and co-infection with other vector-borne diseases was not described. In most cases however, subclinical infections occurs with a prevalence ranging from 3-9% [82,83].

### Canine Leishmania and Trypanosoma

Canine leishmaniasis is caused by protozoa belonging to the genus *Leishmania*. Among the Leishmania species known to infect humans worldwide, only Leishmania tropica and Leishmania donovani are presumed to be anthroponotic [84-86]. There are two forms of leishmaniasis, cutaneous and visceral. Cutaneous leishmaniasis is associated with members of *Leishmania aethiopica*, *L. major*, *L. tropica*, *L. mexicana *and *L. braziliensis *while visceral leishmaniasis is caused by *L. donovani*, and *L. infantum *[63]. Dogs are considered to be the major reservoir for the visceral form of human disease [87]. Sand flies of the genus *Phlebotomus *and *Lutzomyia *are the primary vectors responsible for disease transmission; the infected female sand fly inoculates a vertebrate host with flagellated promastigotes during a blood meal [63]. Leishmaniasis is a slowly progressive disease that can take up to 7 years to become clinically apparent in dogs. However, for reasons that are not well understood, many dogs appear naturally resistant to this parasite and may remain asymptomatic [87]. It is estimated that only 10% of dogs residing in endemic areas actually develop clinical disease [63] with the majority acting as subclinical carriers. Furthermore, up to 20% of infected dogs may mount an adequate immune response and spontaneously recover from clinical illness [88]. Cutaneous lesions are present in up to 89% of infected dogs, with or without overt signs of visceral involvement [87]. However, it should be noted that any animal presenting with apparent lesions should be presumed to have disseminated leishmaniasis because involvement of the integument often occurs late in disease progression [87]. One of the most consistent findings among dogs infected with *Leishmania *spp. is the presence of hyperproteinaemia due to hyperglobulinaemia, often in conjunction with hypoalbuminaemia, while deposition of immune complexes into joints and kidneys results in polyarthritis and glomerulonephritis respectively [87].

Both species of *Leishmania *considered to be anthroponotic are present in India; *L. donovani *the cause of 'kala azar' and *L. tropica*, the cause of cutaneous leishmaniasis. Although sporadic case reports of cutaneous leishmaniasis in dogs from Rajasthan do exist [89], the dog's role as a zoonotic reservoir for these *Leishmania *species has remained largely unexplored. A report of cutaneous leishmaniasis in Rajasthan highlighted the presence of clinical cases in dogs corresponds to the area with human cutaneous leishmaniasis, with 24% (6/25) pet dogs, 21% (17/79) stray dogs and 68.04% (64/95) humans positive for amastigotes (presumably *L. tropica*) from skin lesion smears [90]. Recently, 161 cases of localized cutaneous leishmaniasis were reported in humans from 2001-2003 in the northern state of Himachal Pradesh [91]. Molecular characterization of 10 isolates from humans in this sub-alpine region revealed a unique strain of *L. donovani *in 8 and *L. tropica *in 2 out of the ten patients. The vector and potential reservoir hosts for this novel strain of *L. donovani *remain unknown.

Trypanosomiasis due to *Trypanosoma evansi*, also known as 'surra' in tropical regions of the world, is generally regarded as an important disease of large ruminants and horses. It is transmitted mechanically by tabanid flies (*Tabanus *spp.) and *Stomoxys *spp.. However dogs can also become infected following consumption of the carcass of an infected animal. Trypanosomiasis occurs throughout Asia, although it appears to affect companion animals infrequently [10]. *Trypanosoma evansi *infection in dogs causes a severe disease, with affected individuals showing signs of malaise, fever, generalized oedema, corneal opacity, anaemia, liver enlargement and rapid progression to death. Diagnosis of canine trypanosomiasis is generally made by observation of trypomastigotes in thick or thin blood films, buffy coat smears, or in tissues by mouse inoculation. However, examination by microscopy may under-diagnose the disease because in chronic infection level of parasitemia can be very low [92]. Recently, a TaqMan PCR assay using ribosomal DNA has been develop to detect *T. evansi *and determine the number of organisms present in a blood sample from an infected animals [93]. The parasites have been observed in the blood of clinically normal dogs, suggesting that it does not always cause serious disease and subclinical infection can occur [10].

In a survey conducted at Ludhiana [94], 4.68% (3/64) of dogs were found to be sub-clinically infected through examination of blood smears during the rainy season. These dogs were kept mainly in an area with a considerable population of dairy cattle. Recently, a cattle farmer was diagnosed with trypanosomiasis caused by *T. evansi *in Maharashtra, India [95]. Even though transmission of the disease in this case was speculated to originate from the cattle population, the possibility of dogs becoming another animal reservoir for human infection cannot be dismissed.

## Conclusion

The information regarding CVBD in India is still far from clear. Available literatures imply that filariasis, babesiosis and ehrlichiosis are endemic throughout India, however the identity of these CVBD to a species level remain anecdotal. Despite advances in diagnosis and prophylaxis resulting from extensive research in recent years, most cases and surveys of these diseases in India were diagnosed by morphological observation only, a technique that is limited by its low sensitivity and specificity. Thus, a more comprehensive review of the prevalence of these diseases is still required. A greater understanding of these diseases encompassing both the veterinary clinical aspects and their potential public health significance is also needed in order to better inform veterinarians and pet owners about the risks, prevalence, treatment and control of CVBD. Future studies engaging molecular tools and vector investigation will hopefully help in achieving this aim.

## Competing interests

The authors declare that they have no competing interests.

## Authors' contributions

PAMAR was involved in intellectual content, preparation and writing the manuscript. PJI, MG, GTC and RJT were involved in intellectual input and critical revision of the manuscript for publication. All authors read and approved the final manuscript.
